# Delayed Presentation of Horner’s and Harlequin-Like Symptoms Following Interscalene Peripheral Nerve Catheter Placement

**DOI:** 10.7759/cureus.78070

**Published:** 2025-01-27

**Authors:** Nicholas Wu, Gabrielle Statzer, Akshay Thontakudi, Hamed Sadeghipour

**Affiliations:** 1 Anesthesiology and Critical Care, Sisters of St. Mary (SSM) Health Saint Louis University Hospital, Saint Louis, USA

**Keywords:** catheter migration, delayed-onset horner’s syndrome, harlequin syndrome, interscalene block, local anesthetic

## Abstract

Interscalene peripheral nerve catheters are a commonly performed procedure often used to provide continuous outpatient analgesia following orthopedic procedures. In this case report, we present an interesting case of a patient who received an interscalene catheter following an orthopedic procedure and demonstrated an atypical presentation of combined partial Horner’s and Harlequin-like syndromes evolving more than 36 hours after block placement. Although several case reports in the literature exist describing incidents in which interscalene catheter migration has led to the late onset of complications, it has never before been observed with either this degree of delay or this combination of symptoms. This case emphasizes the importance of recognizing irregular presentations of interscalene block complications secondary to catheter migration.

## Introduction

Brachial plexus peripheral nerve blocks are a well-described regional anesthetic procedure with significant opioid-sparing utility for patients undergoing procedures involving the clavicle, shoulder, and proximal arm. The increasing prevalence of brachial plexus peripheral nerve blocks warrants further investigation of their potential adverse effects. Interscalene blocks (ISBs) are a relatively safe procedure, with common adverse events being phrenic nerve paralysis (nearly 100%) [1], paresthesias (9%) [2], and Horner’s syndrome (6%-12%) [2]. More clinically serious or rare complications of regional blocks have also been documented, including pneumothorax (0.2%-3%) [2] and Harlequin syndrome [3], whose incidence is yet to be estimated in the literature due to their rarity.

Continuous catheter-based interscalene block (CISB) has been explored as an anesthetic option over its single-injection interscalene block (SISB) counterpart. A meta-analysis conducted in 2018 by Vorobeichik et al. found that CISB as an alternative to SISB was superior with regard to several important clinical metrics, including the frequency of postoperative nausea and vomiting, dynamic pain scores, and time to discharge [4]. Vorobeichik et al. also found that CISB reduced average cumulative opioid consumption at 24 hours and 48 hours postoperatively.

While evidence exists supporting the efficacy of CISB over SISB, little data exists discussing the relative drawbacks of CISB. Borgeat et al. reported a lower incidence of all-cause complications in CISB compared to SISB but did not otherwise provide more granular data with regard to incidence rates of the less common complications [2]. Therefore, we sought to shed further light on these rarer complications as well as variance in the timing of their presentations.

## Case presentation

A 25-year-old male American Society of Anesthesiologists grade 2 with a past medical history of epilepsy presented to our hospital with a recurrent right shoulder dislocation and was ultimately scheduled for an open stabilization surgery. On the day of surgery, the patient provided consent for CISB as part of our hospital’s routine protocol for this orthopedic procedure. The patient received 2 mg midazolam before placement of a right-sided interscalene nerve block with catheter placement for surgical analgesia and postoperative pain management. The patient was given an initial bolus of 30 cc bupivacaine 0.5% using an 18-G Touhy needle under ultrasound guidance. A catheter (ON-Q® PainBuster® Catheter, Avonas Medical, Alpharetta, GA, USA) was then placed in-plane in the patient’s right brachial plexus under ultrasound guidance confirming the tip was in the proper position with an injection of 1 mL of air under ultrasound visualization. The placement was uneventful and the position of the catheter was confirmed to be adjacent to brachial plexus roots with the extension of local anesthetic seen in the supraclavicular view. Liquid adhesive (Mastisol, Eloquest Healthcare, Ferndale, MI, USA) was used to keep the catheter in place before it was securely fastened with dressing (Tegaderm, 3M, Saint Paul, MN, USA). No immediate complications were observed. The patient possessed a good sensory and motor block before induction of general anesthesia. The surgery proceeded under general anesthesia and ended without complications. In the postoperative anesthesia care unit (PACU), the catheter was attached to a 600 cc 0.2% ropivacaine On-Q pump and was set at a basal rate of 10 cc/hour per our departmental protocol. The patient did not receive any opioids or pain medications besides the block in the PACU. The patient reported good pain control and was discharged that evening uneventfully.

Routine anesthesia telehealth follow-up was conducted on postoperative day (POD) one according to our departmental policies. During the call, the patient noted diffuse swelling of the right arm in the morning (approximately 20 hours after CISB placement), consistent with expected postoperative swelling. At that time, the On-Q catheter was still set at a rate of 10 cc/hour and the patient reported the catheter dressing was clean and intact. The patient reported that though the catheter was still providing pain relief at that time, it had become less effective. He denied any motor dysfunction or symptoms otherwise. On the night of POD one, the patient noted significant flushing and warmth confined to the left face as well as continued right arm swelling distal to the catheter. The patient did not seek medical attention at the time and slept without issue. The morning after that, more than 36 hours after initiation of the CISB, the patient woke up with impaired vision due to significant ptosis of the right eyelid. The previous flushing and warmth on the left face had also persisted overnight. There were no signs of left arm weakness. The patient decided to immediately remove the catheter at that time. On telehealth follow-up later that day, the patient informed the anesthesiology team that within one hour after catheter removal, his symptoms completely subsided on both sides of the face, and he did not experience any further complications after removal. He denied ever having any symptoms of anhidrosis and did not observe any miosis. Pain scores were unchanged from the day prior. The patient’s sensation and motor function were confirmed to be at baseline, and the patient denied ever having this type of reaction before. Interestingly, during a similar surgery three months later on the left shoulder, the patient received another ISB with catheter placement on the left side with ropivacaine and experienced immediate ipsilateral Horner’s syndrome but no symptoms suggestive of Harlequin-like syndrome.

## Discussion

Horner’s syndrome is a known transient complication of ISB in which patients typically present with a constellation of symptoms due to the local spread of anesthetic to the cervical sympathetic chain. The resulting ipsilateral stellate ganglion block classically leads to ptosis, anhidrosis, and miosis. Hoarseness may also occur due to the cervical sympathetic chain’s proximity to the recurrent laryngeal nerve. Although Horner’s due to ISB typically presents immediately or shortly after initiation of the block, Alzahrani et al. reported a patient with delayed Horner’s syndrome eight hours after CISB [5]. No cases of CISB have been documented with a degree of delayed presentation greater than eight hours.

Conversely, Harlequin syndrome is a less common dysautonomic sequelae of ISB with an unknown rate of incidence. Harlequin syndrome classically presents with pallor and anhidrosis ipsilateral to the insult accompanied by contralateral facial flushing, hyperemia, and sweating with a clear demarcation in the facial midline. In the literature, an incomplete constellation of these symptoms is typically referred to as subclinical Harlequin syndrome or Harlequin-like syndrome. Harlequin syndrome has often been reported in various surgical and anesthetic contexts, including thoracic epidural placement, intercostal block, and ISB. The underlying pathophysiology is theorized to be linked to unilateral blockade via injury or compression of the T2-T3 nerve fibers supplying the face, with exaggerated sympathetic thermoregulatory vasodilation as a compensatory mechanism on the contralateral facies. Despite its striking appearance, the clinical course of Harlequin’s is relatively benign and typically resolves with cessation of anesthetic delivery, but can be alarming to patients and providers alike. To date, there are only three reports of Harlequin syndrome associated with ISB recorded in the literature. Two of these reports are associated with SISB, while the other occurred in the context of CISB [6-8]. All three incidents occurred within one hour of emergence from general anesthesia, and only the CISB occurred simultaneously with Horner’s syndrome.

Our case represents a novel finding in that our constellation of symptoms, namely, partial Horner’s and Harlequin-like syndrome occurring in tandem, has never been documented with this degree of delayed onset in any ISB. Our patient exhibited the ipsilateral ptosis associated with Horner’s syndrome, as well as the contralateral flushing and warmth consistent with Harlequin-like syndrome. In the aforementioned case of a continuous interscalene nerve block with partial Horner’s and Harlequin-like syndrome occurring immediately after placement, Cheng et al. discovered that the interscalene catheter’s tip had migrated adjacent to the thyrocervical artery via ultrasound despite initial confirmation of correct placement at the time of the block [8]. Considering the patient history and time course, we posit that the delayed presentation seen in our patient is a similar consequence of interscalene catheter migration occurring more than 36 hours after initial placement. The fact that virtually all reported single-injection ISB-related incidents of Horner’s syndrome occur within one hour of administration lends further credence to our proposed mechanism for delayed presentation in this patient. Postoperative migration of catheter tips has been associated with worsened pain scores, likely as a consequence of diminished anesthetic delivery and effect. In our case, it is worthwhile to note that the patient was still experiencing some analgesia at the time of symptom onset.

Although there is limited data regarding the frequency of catheter migration, with Aoyama et al. estimating its incidence between 33.3% and 40.7% at 24 hours [9], these complications remain an important consideration in the placement of interscalene catheters that anesthesia personnel must be able to identify. Migration can occur either as a result of extensive catheter threading or dislocation secondary to patient movement. Though uncommon, intrathecal migration of CISB has resulted in sudden death in at least one patient more than six hours after catheter placement [10]. Therefore, the standard of care should include pre-emptively educating patients on signs of possible migration, such as flushing or vision changes, to facilitate early detection and prevention of adverse outcomes. Alzahrani et al. further proposed that catheters should be activated immediately after insertion to help guide earlier identification of adverse complications [5]. Ultrasound visualization and appropriate fastening after catheter placement is also an essential step in preventing migration-related complications but is nevertheless not a perfect safeguard given the potential for immediate migration after placement. Once discovered, management of symptomatic catheter migration involves prompt removal of the catheter.

We acknowledge the limitations of our case study, most specifically with regard to our inability to visualize catheter positioning via ultrasound at the time of symptom evolution as the patient removed the catheter independently, as well as our inability to directly confirm the clinical manifestations reported by the patient on physical examination.

## Conclusions

Ultimately, interscalene nerve catheters possess significant analgesic utility, but physicians and patients should be cognizant of the variable side effects including Horner’s and Harlequin-like syndrome and maintain a high level of clinical suspicion for their possibility. While there has been literature reporting each clinical syndrome in isolation, this article represents the first time both presentations have been found simultaneously in such a delayed fashion. Their late appearance highlights the potential for more devastating complications with late presentation. Although the majority of complications associated with interscalene catheters are transient and not clinically worrisome, the evolution of symptoms can cause alarm for both patient and provider alike. This should not, however, preclude the use of brachial plexus blocks given their demonstrated utility in opioid-sparing regimens as well as increased patient satisfaction. Further research weighing the relatively lesser efficacy of single bolus ISBs against the possibility of catheter migration in continuous blocks is likely warranted.
